# Concentrated Vegetable Medicines

**Published:** 1876-05

**Authors:** A. G. Stallnaker

**Affiliations:** Walton, West Virginia


### CONCENTRATED VEGETABLE MEDICINES.

By A. G. STALLNAKER, M.D., Walton, West Virginia.

*Concentrated Tincture of Diascorea*, from *Diascorea Villora*, commonly called Wild Yam, is as near a specific for bilious colic, cholera morbus, as any agent I have ever tried. In infantile colic, or intestinal irritation, one or two drops in a teaspoonful of milk, will relieve without constipating, and has never failed to give relief in my hands. The dose can be repeated as often as necessary.

*Concentrated tincture of Stillingia*, from *Stillingia Sylvatica*, commonly called Queen's Delight, as an alterative in syphilis, scrofula, or in any case demanding a positive agent, is invaluable. While physician on the Western Division of the Chesapeake and Ohio railroad, I treated successfully a large number of cases of syphilis, and the fact that they were principally negroes, who were not as cleanly as possible, is an additional proof of its curative power. The following prescription is my usual mode of giving it:

R. Concentrated tincture stillingia, drachms j
" " phytolacca.
" " podophyllum.
" " xanthoxylum, aa drachms ss.
Pure water....................................drachms viij.

Mix. S: Teaspoonful twice a day. Apply strong solution of chloride of zinc to the sores. If there be much pain two grains of sulphate of morphia can be added to above. In cases of bone complication, I add one drachm of phosphate of iron, as I think it assists nature very materially in throwing off the old and forming new bone.

*Concentrated tincture of Phytolacca*, from *Phytolacca Decandra*, commonly called poke root, is my next best vegetable alterative, and is very decided in its action. As a remedy in rheumatism, it has proven very efficacious. I sometimes use it in connection with concentrated tincture of xanthoxylum, in the proportion of six drops of the former to four of the latter, three times a day. If a liniment is indicated, I use chloroform and tincture of capsicum. Five drops of concentrated tincture of gelseminum at bedtime has proven very beneficial.

*Concentrated tincture of Podophyllum*, commonly called May-apple root, is too well known to require me to say more than that it should never be used alone, for when so given, it has a most griping, sickening, cathartic effect, but is thorough and definite in its action on the liver. I once took nine grains of pulverized podophyllum to test it, and am perfectly satisfied as to its power. In combination with concentrated tincture of leptandria and tincture of capsicum—one drop phodophyllum, two drops leptandria and one drop capsicum—it will meet the most reasonable expectation of any practitioner. Its use is indicated in every ease in which calomel is given, and owing to the peculiar idiosyncrasy of some about taking calomel, the above combination is the best vegetable substitute.

*Concentrated tincture of Hydrangea*, commonly called Seven Bark, as a remedy for gravel, will, in a few years, be in every physician's office. In the treatment of cystitis, or any form of inflammation of the bladder, painful micturition, etc., it has been a great aid to me. In come cases I find great benefit when I use equal parts of concentrated tincture of populous with it. One beauty of these concentrated tinctures is, that they can be carried in a small space, are easily and quickly dissolved in the stomach, and they can be combined with most any other medicine.

*Concentrated tincture of Veratrum*, from *Veratrum Viride*, commonly called American hellebore, is a remedy not only of great value in fevers of all kinds, by controlling the frequency and force of the pulse, but it acts also on the secretions of the skin and kidneys. For an adult I add ten drops of the tincture to two ounces of water, and give a teaspoonful every hour as long as needed. It is an emetic in large doses, and not considered safe if used heroically. In the treatment of erysipelas, I desire particularly to call the attention of the profession to its action. In cases in which muriated tincture of iron and iodine have failed to relieve, the veratrum has always acted quickly and delightfully. Mix a teaspoonful in two ounces of water, and apply every two or three hours as the case demands. I have just relieved a patient who had been using sugar of lead, carbolic acid and iodine with ill effect. Of course you must expect a return of the disease until you get the blood purified by the use of alteratives, as I regard it as proceeding entirely from that cause. A few weeks ago, I was called in to see a child with scald head, or eczema might be more appropriate; I ordered the application as above described, and the next morning the baby's head had healed up very much. An incrustation formed and fell off, and the child got well. Its head and face was nearly one entire sore, and the little sufferer was very fretful.

*Concentrated tincture of Hydrastis*, from *Hydrastis Canadensis*, commonly called golden seal, or yellow puccoon, acts on the mucous surface most certainly, and its use cannot be mistaken. In leuccorrhœa and ophthalmia I often use no other medicine, though I frequently add twenty grains of sulphate of zinc to the drachm of hydrastis, and dilute to suit the exigencies of the case. I hope others will try this on any form of sore eyes and report to the *Monthly*. As a bitter tonic, in conjunction with pure whisky it is very strengthening in weak conditions of the stomach. Last summer, while riding up Wills Creek, Kanawha county, a Mrs. Strickland asked me to come in and see her little child, stating that it "had lost the sight of one of its eyes, and the other one was very weak." The girl was apparently healthy otherwise, about 9 years old, and had not been able to see out of the eye for some months, and felt that the other eye was going likewise. Upon examination, I found a thin, clear, flimsy substance had grown over the entire ball, but the hydrastis and zinc took it off in a short time—three days, I believe—and the child is now blessed with good eyesight.

*Concentrated tincture of Xanthoxylum*, from *xanthoxylum fraxineum*, commonly called prickly ash, is a lasting stimulant, tonic and alterative. In rheumatism especially, whether chronic or acute, it is nearly certain to afford relief, and has even proven of value in the crude form, by adding two hands' full of bark to a pint of whisky. In the concentrated state you know what doses you are giving, and can combine it with whisky or anything else and know what you are doing. Of all the remedies in the materia medica I prize this the most highly for rheumatism.

I wish to distinctly state that I have never found the same satisfaction from this class of drugs from any other house as that of Messrs. B. Keith & Co., of New York City, whose preparations I have used for the last ten years. I am well aware "antiquity has not spread her dusty mantel" over these remedies, but those who have only used the agents in a crude state can little appreciate the reliability of the concentrated tinctures. They might as well take a pint of rye and pour hot water over it and call it whiskey, as to use the crude substances and pronounce an opinion as to the medicinal virtues of these tinctures. I reiterate that, as domestic remedies, they have not proven of much utility, but as officinal medicines, they are of incalculable value.— *Virginia Medical Monthly.*

